# Diabetes in Relation to Serum Levels of Polychlorinated Biphenyls and Chlorinated Pesticides in Adult Native Americans

**DOI:** 10.1289/ehp.10315

**Published:** 2007-07-17

**Authors:** Neculai Codru, Maria J. Schymura, Serban Negoita, Robert Rej, David O. Carpenter, The Akwesasne Task Force on the Environment

**Affiliations:** 1 Department of Epidemiology and Statistics, School of Public Health, University at Albany, Rensselaer, New York, USA; 2 New York State Department of Health, Albany, New York, USA; 3 Mohawk Nation at Akwesasne, Hogansburg, New York, USA; 4 Wadsworth Center, New York State Department of Health, Albany, New York, USA; 5 Department of Biomedical Sciences, School of Public Health, University at Albany, Albany, New York, USA; 6 Institute for Health and the Environment, University at Albany, Rensselaer, New York, USA

**Keywords:** BMI, DDE, fasting glucose, hexachlorobenzene, mirex, polychlorinated biphenyls

## Abstract

**Background:**

Recent research suggests that diabetes, a condition whose incidence is increasing, is associated with exposure to polychlorinated biphenyls (PCBs) and chlorinated pesticides.

**Objectives:**

We investigated the potential association between diabetes and serum levels of PCBs, dichlorodiphenyldichloroethylene (DDE), hexachlorobenzene (HCB), and mirex in a cross-sectional study of an adult Native-American (Mohawk) population.

**Methods:**

Through a standardized questionnaire we collected demographic, medical, and lifestyle information from 352 adults, ≥30 years of age. We collected fasting serum samples that were analyzed for 101 PCB congeners, DDE, HCB, and mirex along with fasting glucose, triglycerides, and cholesterol. Participants who had fasting-glucose values > 125 mg/dL and/or who were taking antidiabetic medication were defined as persons with diabetes. We conducted logistic regression to assess the potential association between organochlorine serum levels and diabetes, while controlling for the potential confounding variables of age, body mass index (BMI), smoking, sex, and serum lipid levels. Organochlorine serum levels were categorized in tertiles, and the lowest tertile was used as the reference category.

**Results:**

The prevalence of diabetes was 20.2%. The odds ratio (OR) of having diabetes for participants in the highest tertile of total PCB concentration compared with the lowest tertile was 3.9 (95% confidence interval, 1.5–10.6). The corresponding ORs for DDE and HCB were even higher. Elevated serum mirex was not associated with diabetes. After adjustment for other analytes, the OR for HCB remained significant, whereas ORs for PCBs and DDE remained elevated but not statistically significant. In contrast, after adjustment for other analytes, the OR for mirex became statistically significant and indicated an inverse association.

**Conclusions:**

In this study of adult Native Americans, elevated serum PCBs, DDE, and HCB were positively associated with diabetes after controlling for potential confounders, whereas a negative association was observed for mirex.

Diabetes is one of the most prevalent chronic diseases in developed countries, conferring a significant burden in terms of medical complications and health-care costs. Between 1980 and 2004, the number of Americans with diabetes increased from 5.8 million to 14.7 million. In 2004 alone, there were approximately 1.4 million new diagnoses of diabetes in American adults (18–79 years of age) [Centers for Disease Control and Prevention (CDC) 2005]. Incidence and prevalence of diabetes vary by age, ethnicity, and socioeconomic factors and are, in general, higher in Native Americans (CDC 2003a). Known risk factors for diabetes include obesity, genetic susceptibility, hyperinsulinemia (a marker for insulin resistance), sedentary lifestyle (Warram and Krolewski 2005), and cigarette smoking (Rimm et al. 1995; Will et al. 2001).

Polychlorinated biphenyls (PCBs) were produced for use in various industries until the late 1970s when their production was banned. By then, large quantities of PCBs had been released into the environment. They are persistent substances both in the environment and in biota, and they bio-accumulate and biomagnify in the food chain. Once in the human body they persist for long periods, accumulating in adipose tissue and in the lipid component of serum.

The Mohawk Nation at Akwesasne is a Native American population residing along the St. Lawrence River that separates New York State from Ontario and Quebec (Canada). Mohawks are traditionally a fish-eating community. There are three aluminum foundries just upriver from the reservation, namely the General Motors Central Foundry Division (a National Priority List site) and plants operated by Reynolds Metal and the Aluminum Company of America (ALCOA) (Hwang et al. 1993). PCBs (primarily Aroclor 1248) were used as hydraulic fluids at all three facilities. PCBs that leaked were washed into the St. Lawrence River and its tributaries. Via the air, soil, and water, PCBs contaminated the environment and local flora and fauna, and entered the food chain. Although they are only modestly elevated, PCB levels in Mohawk breast milk (Hwang et al. 1996) and serum (Fitzgerald et al. 2004) have been correlated with rates of consumption of local fish, even though fish consumption has declined in recent years after issuance of advisories.

Recent studies have reported an association between exposure to organochlorines and impaired blood-glucose regulation and diabetes. Several epidemiologic studies (Calvert et al. 1999; Pesatori et al. 1998; Vena et al. 1998) have shown that dioxin exposure is associated with elevated rates of diabetes and dysglycemia. U.S. Air Force veterans of Operation Ranch Hand, who applied Agent Orange in Vietnam, were exposed to dioxin. Exposure was associated with an elevated incidence of diabetes and was inversely associated with the length of time to diabetes onset (Henriksen et al. 1997). Cranmer et al. (2000) reported that dioxin exposure resulted in hyperinsulinemia and insulin resistance, and Fierens et al. (2003) reported significant odds ratios (ORs) of 5.1, 13.3, and 7.6 for risk of diabetes in relation to top decile concentrations of dioxins, coplanar PCBs, and 12 PCB markers. Longnecker et al. (2001) reported that pregnant women with diabetes had higher PCB levels than did nondiabetic pregnant women. Radikova et al. (2004) reported that PCB concentrations in a Slovak population were associated with elevated levels of blood glucose. Vasiliu et al. (2006) found a linear association between PCB serum levels and diabetes incidence in a large cohort in Michigan, and Lee et al. (2006) found a strong dose–response relationship between serum concentrations of six persistent organic pollutants [PCB-153, two dioxin congeners, oxychlordane, dichlorodiphenyldichloroethylene (DDE), and *trans*-nonachlor] and diabetes. Everett et al. (2006) reported an association between serum levels of both PCB-126 (a dioxin-like PCB) and *p,p*′-DDT (dichlorodiphenyltrichloroethane). DDE, the major metabolite of DDT, has previously been reported to be associated with diabetes (Glynn et al. 2003; Rylander et al. 2005), and hexachlorobenzene (HCB) was also linked to diabetes in cross-sectional studies (Glynn et al. 2003). Lee et al. (2007) have expanded their study to analyze the data from the sum of four dioxin-like PCB congeners and five nondioxin-like congeners, and report that the dioxin-like congeners showed the strongest relationship with diabetes.

The present study was designed to investigate whether a relationship exists between diabetes and serum levels of total PCBs, two single PCB congeners, and the chlorinated pesticides DDE, HCB, and mirex in adult Mohawks.

## Materials and Methods

Mohawk adults ≥30 years of age who resided at or near Akwesasne for at least 5 years were eligible for this study. Recruitment took place between 1995 and 2000, and sampling was performed on a household basis. A listing of all households was constructed by Mohawk field staff with the aid of detailed maps of the reserve. Staff members drove through assigned sections of the reserve and systematically reviewed all structures and cataloged housing units. Multiple-family dwelling units were subdivided into individual households. Known Mohawk housing units in the vicinity of Akwesasne but off the reservation were added to the list. Once this list was completed, a simple random sample of households was selected. Selected households were visited by project staff, who determined the composition of the household and study eligibility. One eligible adult per household was invited to participate. We were able to ascertain household composition for 68.1% of selected households and enrolled 401 (65.3%) eligible adults into the study. The final study population consisted of 352 participants for whom we had all relevant blood measurements.

Written informed consent was obtained from the participants. All participants were administered a core interview that included demographic information and questions on diet, residential and occupational exposures and education. The standardized questionnaire, administered via an in-person interview, included open-ended questions on participants’ medical conditions and medications. Medication used in this population for glucose regulation included glyburide (34 participants), insulin and its analogues (13 participants), metformin (18 participants), and troglitazone (2 participants).

Blood samples were obtained by venipuncture between 0700 and 1030 hours after overnight fasting in 5-mL collection tubes for analysis of glucose, triglycerides, and cholesterol and a separate sample (10 mL whole blood for ~ 5 mL serum) for analysis of 101 PCB congeners, DDE, HCB, and mirex. The blood samples were allowed to clot at room temperature for 1 hr, then centrifuged and the serum removed. Serum for both analyses were then stored at –80°C on-site until being transported on dry ice to the laboratories for analysis.

Serum glucose and lipid concentrations were measured in the New York State Department of Health Laboratory (Wadsworth Center) on a Hitachi 911 analyzer (Roche Diagnostics, Indianapolis, IN) using the hexokinase and glucose-6-phosphate dehydrogenase coupled method for glucose (Kunst et al. 1984), and cholesterol esterase and oxidase, as well as peroxidase for total cholesterol (Allain et al. 1974). Triglycerides were determined by a glycerol kinase-based procedure that corrects for free glycerol in the specimen (Kohlmeier 1986), as recommended by the National Cholesterol Education Program Working Group on Lipoprotein Measurement (Stein and Myers 1966). The facility is approved by the Clinical Laboratory Improvement Amendments and is a member of the CDC reference laboratory network for lipid measurements (Myers et al. 2000). Total serum lipids were calculated using the “short” formula proposed by Phillips et al. (1989) and recently validated by the same group (Bernert et al. 2007): Total lipids (mg/dL) = 2.27 × total cholesterol (mg/dL) + triglycerides (mg/dL) + 62.3.

PCB analysis was performed in the Exposure Assessment Laboratory of the University at Albany as described by DeCaprio et al. (2000). The ultratrace analytical methods used dual-column gas chromatography with electron-capture detection to measure 92 analytical peaks that represent 83 single PCB congeners and 18 congeners as pairs or triplets, for a total of 101 PCB congeners, plus DDE, mirex, and HCB. Results are reported as both wet weight and lipid-based values. Lipid-based values were determined by dividing the wet weight value by total serum lipids as calculated above and then multiplying by a factor of 10^5^ for unit adjustment (nanograms of toxicant per gram lipid). Values below the method detection limit (MDL) were set to zero.

Diabetes was defined as having a fasting-glucose value > 125 mg/dL [American Diabetes Association (ADA) 2003a, 2007] or taking physician-ordered antidiabetes medication. We included known risk factors, age, obesity (indicated by the body mass index; BMI), and smoking (defined as a categorical variable so that participants who smoked at least 100 cigarettes over their lifetime were classified as smokers and all others as non-smokers). Age was included as a categorical predictor, dichotomized at 45 years. We chose 45 years for several reasons: 45 is the recommended age to begin diabetes screening (ADA 2003b); it is close to the median age of our study population; and we found that dichotomizing age at 45 years provided optimal control for confounding. BMI (in kilograms per square meter) was categorized as proposed by the CDC (2005): < 25, 25–29.9 (corresponding to overweight), and ≥ 30 (corresponding to obesity). BMI < 25 was the reference category.

To account for potential risk factors and confounders (serum lipid levels, age, BMI, sex, smoking history) we conducted logistic regression. The outcome of interest was dichotomous (yes/no). The decision to introduce smoking in the analysis was based on the fact that smoking is associated with both diabetes (as a risk factor) and toxicant values. Deutch et al. (2003) reported that smoking status was a determinant of the blood organochlorine levels in Greenland natives. We also considered sex to be a potential confounder because of the differences in fat content, distribution, and modes of excretion (e.g., lactation in women) between the sexes. Therefore, organochlorine dynamics may be different in females than in males.

### Statistical analysis

The association between diabetes and established risk factors was initially analyzed through bivariate analysis of each factor separately. We also performed bivariate analysis for the total PCBs, two individual congeners, and three organochlorine pesticides. The toxicants were grouped in tertiles of exposure, with the lowest one serving as the reference (comparison) group.

Logistic regression was used for all multivariable analyses. We first estimated the association of diabetes with the serum wet-weight concentration of total PCBs, PCB-153, PCB-74, DDE, HCB, and mirex, one analyte at a time, while adjusting for age, sex, BMI, smoking status, and estimated total lipid concentration. We then measured the association of total PCBs, DDE, mirex, and HCB while simultaneously adjusting for all analytes and the above-mentioned diabetes risk factors. Subsequently, we measured the association of diabetes with the serum concentration of PCB-153 and PCB-74 while simultaneously adjusting for the serum concentration of the chlorinated pesticides and diabetes risk factors. Finally, we replicated the analyses using lipid-standardized concentration values. In the later models, all estimates of association were adjusted for the diabetes risk factors minus the estimated total lipid concentration. All analyses were conducted using SAS software (version 8.2, SAS Institute Inc, Cary, NC).

## Results

Tables 1 and 2 show the characteristics of the final study population for whom all blood measurements were available. These tables present age, BMI, and fasting-glucose and serum lipid levels as well as antidiabetes medication. Women comprised almost 62% of participants, and about three-fourths of the participants were smokers. Seventy-one participants (20.2%) were diabetic, according to our criteria. All participants taking medication for glucose regulation also reported they had been diagnosed with diabetes, although some with elevated fasting-glucose levels were not taking glucose regulatory medication.

Table 3 shows wet weight and lipid-adjusted levels of total PCBs, two individual PCB congeners (PCB-153 and PCB-74), and the three pesticides, and gives the MDL and percentage of samples above the MDL for the PCB congeners and the pesticides. Also shown are the minimum, 33%, 50%, 67%, and maximum values. Total wet weight serum PCB levels ranged from 0.51 to 48.32 ppb; in 95% of the subjects, the total PCB serum levels were < 13 ppb. Lipid-adjusted total PCB values had a median level of 579.8 ng/g lipid (range, 84.8–7110.1 ng/g lipid).

Table 4 presents results showing the association between diabetes and total serum PCBs, mirex, DDE, and HCB by tertile, adjusted for sex, age, BMI, and smoking status. Also shown are results after concurrent adjustment for each of the other analytes. We observed a significant association for highest versus lowest tertile of both wet weight (OR = 3.90) and lipid-based (OR = 3.29) PCBs and diabetes, but this relationship was less after concurrent adjustment for the pesticides. Mirex showed no relationship with diabetes when other analytes were not included in the model, but a statistically significant inverse association was observed at the highest tertile after controlling for the other analytes (OR = 0.3). There were statistically significant associations between diabetes and both DDE (OR = 6.4) and HCB (OR = 6.2) at the highest tertile. The relationship remained statistically significantly elevated for HCB after adjustment for PCBs, mirex, and DDE, whereas that for DDE remained elevated but not significant after adjustment for other contaminants.

Table 4 also shows similar data for two individual PCB congeners, PCB-153 [the congener present in this population at the highest concentration, and the single PCB congener reported by Lee et al. (2006)], and PCB-74, a mono-*ortho* congener previously reported to be most closely related to rates of fish consumption in this population (Fitzgerald et al. 2006). We found a significantly increased risk of diabetes in relation to the highest tertile (compared with the lowest tertile) for lipid-based PCB-153 and an even higher risk for both wet weight and lipid-based PCB-74 when not adjusted for the levels of pesticides. After concurrent adjustment for the pesticides, the ORs were lower, and the OR for PCB-74 almost reached statistical significance.

## Discussion

Although diabetes has not usually been considered to be an environmentally induced disease, we have found a significant association between serum PCB and pesticide levels and diabetes in an adult Native-American population after adjustment for age, BMI, serum lipid levels, sex, and smoking. Although these results do not establish cause and effect, there is a growing body of evidence that environmental exposure to persistent organochlorine compounds is associated with elevated incidence of this disease. Elevated incidence of diabetes has been demonstrated following dioxin exposure in Seveso, Italy (Bertazzi et al. 1998; Pesatori et al. 1998). Vena et al. (1998) reported similar findings in a large study of workers exposed to dioxins during production of phenoxy herbicides and chlorophenol. Cranmer et al. (2000) found that plasma insulin concentrations were elevated in individuals who had elevated levels of dioxin, and they concluded that dioxin exposure leads to insulin resistance. The studies by Longnecker et al. (2001), Radikova et al. (2004), and Vasiliu et al. (2006) show dose-dependent relationships between diabetes or fasting-glucose levels and PCBs. Kouznetsova et al. (2007) reported elevated rates of hospitalization for diabetes among individuals living near hazardous waste sites, particularly if those sites contain persistent organic pollutants. Perhaps most compelling are the recent reports of Lee et al. (2006, 2007) and Everett (2006), who demonstrated dose–response relationships between serum concentrations of different organochlorine compounds and the prevalence of diabetes. Lee et al. (2006) included PCB-153 (one of the phenobarbital-inducer PCBs), and DDE, as well as two dioxins and two other pesticides. In an editorial concerning this publication, Porta (2006) noted that Lee et al. (2006) did not find an association between obesity and diabetes in individuals with nondetectible levels of organochlorines. This raises the surprising possibility that the real relationship to diabetes is with organochlorine levels, and that the apparent relationship with obesity simply reflects greater consumption of animals fats. Everett et al. (2006) found elevated ORs for diabetes with both PCB-126 (a dioxin-like congener) and *p,p*′-DDT, but not with a hexachlorodioxin. In the most recent analysis of Lee et al. (2007), the relationship to diabetes was much larger for the sum of four dioxin-like PCBs than for the sum of three dioxins, the sum of three furans, the sum of five nondioxin-like PCBs, or the sum of four organochlorine pesticides. In that article, Lee et al. considered PCB-74 to be a dioxin-like congener even though it has not been assigned dioxin toxic equivalents. However, the relationship they observed is consistent with our observations that PCB-74 showed a stronger relationship to diabetes than did PCB-153, which is one of their nondioxin-like congeners.

With respect to HCB, our results are consistent with those reported by Glynn et al. (2003) and Langer et al. (2007). Glynn et al. (2003) found a significantly higher concentration of HCB in women with diabetes than in women without the disease. Langer et al. (2007) reported higher proportions of impaired fasting glucose among subjects from high pollution areas with high serum concentrations of PCB, DDE, and HCB.

We have not found any published reports on the relationship between mirex concentration and diabetes or impaired glucose regulation in humans. Rogers et al. (1984) reported a steep decrease in plasma glucose levels in mirex-treated rat fetuses. However, Ervin and Yarbrough (1985) found no effect of mirex on plasma glucose levels of hypophysectomized rats. Keller et al. (2004) observed a statistically nonsignificant negative correlation between the mirex concentration in adipose tissue of live sea turtles and plasma glucose, as well as a negative association between whole blood mirex concentration and plasma glucose. Our observation that mirex concentrations were inversely related to diabetes is therefore of interest.

The present study has several limitations. Only single measurements were made of both fasting-glucose level and levels of serum PCBs and pesticides. Although participants were instructed to fast overnight before providing blood samples, it could not be objectively confirmed that they did so, and glucose can vary significantly in the nonfasting state. If some participants did not fast as instructed, measurement bias could affect our findings. However, this bias is likely to be nondifferential because there is no reason to suppose that participants with a higher toxicant burden would have been preferentially more inclined not to fast. Although the associations are significant and the ORs are high, the 95% confidence intervals are large. This may be due to the limited number of participants who had an outcome in the lowest tertile, which was the reference category of exposure.

Our method for measurement of serum PCBs gives information on 101 congeners, but it does not include some of the most potent dioxin-like congeners, such as PCB-126. Therefore, we have incomplete information on the dioxin-like activity. We do not have direct measurement of total serum lipids, only of total cholesterol and triglycerides, with application of the formula developed by Phillips et al. (1989) to estimate total serum lipids. Although widely used, this formula was extrapolated from a study with a relatively small number of participants who differed from our study population in ethnicity, sex, and age distribution.

The cross-sectional design of our study does not permit an assessment of the temporality of events; that is, we cannot know whether diabetes results from elevated toxicant levels or vise versa. Longnecker (2006) raised the possibility that pharmacokinetic variability may explain the associations observed between organochlorines and diabetes at background levels of exposure. He cited the lack of positive findings from occupational cohort studies in which individuals were exposed to much higher toxicant concentrations and suggested that at background levels serum concentrations might reflect clearance factors that are related to risk of diabetes through diet or other innate factors in addition to intake.

There are also major strengths to our study. The outcome definition was comprehensive on the basis of fasting plasma glucose values as well as information on the taking of antidiabetes medication. It is notable that all of the participants who were under anti-diabetes treatment responded that they had been diagnosed with diabetes, which suggests that misclassification of disease was not an important issue. Our organochlorine determinations were performed at one time point using the same analytical methods, which minimizes the potential for measurement bias. Our analytical method monitors more PCB congeners than previous investigations, as well as three pesticides. The relationships observed were similar whether contaminant levels were expressed as wet weight or on a lipid basis, and demonstrate strong consistency.

We confirmed the information on the diabetes diagnosis by the recollection of anti-diabetes treatment, as well by blood-glucose measurements. Logistic regression analysis considering different outcome definitions (having diabetes as diagnosed by a physician, being on antidiabetes medication, or having fasting plasma glucose values > 125 mg/dL, each assessed separately) yielded similar findings in terms of significant association between increased organochlorine exposure and occurrence of diabetes.

In our analysis we controlled for other known or possible risk factors for diabetes. As expected, age is a significant risk factor. BMI also proved to be a significant risk factor, which is consistent with previously reported associations between diabetes and obesity [Hu et al. 2001; Kriska et al. 2003; but see Porta (2006)]. Total serum lipid levels were modestly related to risk of diabetes. Smoking has been reported to be a risk factor for diabetes (Rimm et al. 1995; Will 2001). However, smoking, defined as having smoked at least 100 cigarettes in the lifetime, was not found to be significantly associated with diabetes. There was no significant difference by sex, which is consistent with the finding in national studies (CDC 2003b). We did not control for diet, exercise, or physical activity.

Diabetes prevalence in our study population (22.4% in men and 18.4% in women) was slightly higher than that reported for other Native-American and Alaska Native populations adjusted for age (CDC 2003a; Burrows et al. 2000). In a previous study of clinic patients from the same community, Negoita et al. (2001) found a prevalence of diabetes ranging from 2.9% in subjects 30–44 years of age to 21.3% in those > 75 years of age. We found a higher proportion of persons with diabetes across all age groups, which is partially explained though the identification of individuals with elevated blood-glucose levels not previously diagnosed with diabetes.

Because PCBs are very persistent in the human body, fasting serum levels provide some indication of lifetime exposure, even though some congeners are more persistent than others. Although the serum PCB levels in this population (mean of 5.0 ppb) are somewhat higher than in the general population that does not have particular exposure [reported by the Agency for Toxic Substances and Disease Registry (ATSDR) (2000) to be 0.9–1.5 ppb], the range of PCB levels (0.51–48.32 ppb) in adult Mohawks includes background levels found in the general population. For comparison on the basis of a single congener, Lee et al. (2006) reported the concentration of PCB-153 in their study to be 36.7 ng/g lipid in the 25th–50th percentile, and 60.2 ng/g lipid in the 50th–75th percentile, whereas in our Mohawk population the values were 78.3 ng/g lipid at the 50th percentile and 104.4 ng/g lipid at the 67th percentile. The demonstration of the association between levels of total PCBs, DDE, and HCB with diabetes (almost certainly type 2) is consistent with the results of previous investigations and provides additional evidence that this relationship occurs among different ethnicities and populations.

We found statistically significant elevations in risk of diabetes for total PCBs, the two PCB congeners reported separately, and DDE and HCB. The most elevated ORs were found for HCB, both before and after adjustment for other analytes. Although the lower bound of the 95% confidence limit after adjustment for other analytes did not exceed .0 for total PCBs, PCB-74, PCB-153, and DDE, the ORs were elevated. However, it must be recognized that all PCBs and chlorinated pesticides are fat-soluble substances, which means that they migrate together. Therefore, caution must be taken in drawing conclusions on the question of which substances are more important in explaining the relationships observed.

The biochemical mechanisms underlying relationship between diabetes and serum levels of organochlorines are still uncertain. In animal studies, morphologic changes have been reported in the structure of beta cells in pancreas upon PCB exposure (Kimbrough al. 1972; Wassermann et al. 1975), and altered expression of gluconeogenic enzymes were found in rat liver (Boll et al. 1998). HCB has been reported to disrupt the gluconeogenic pathways in animal models (Mazzetti al. 2004), and it is possible that other organochlorines have similar actions. Other potential mechanisms involve the organochlorine impacts on the immune system (Langer al. 2002), as well as a dioxin-like action on insulin regulation, an action that may be mediated through sex-hormone binding globulin, as suggested by Michalek et al. (1999). PCBs induce several different cytochrome P450s in the liver and other tissues (Bandiera 2001); this results in unique patterns of gene induction (Vezina et al. 2004). We suspect that if these relationships are ultimately found be causative, the explanation will come from the gene induction that results from exposure to substances that are metabolized by cytochrome P450s.

## Conclusion

In this cross-sectional study, serum concentrations of total PCBs, two single PCB congeners, DDE, and HCB were positively associated with an elevated incidence of diabetes in an adult Native-American population. These findings are consistent with the hypothesis that exposure to organochlorine compounds increases the risk of developing diabetes. A negative association was found between the serum concentration of mirex and diabetes. This finding has not been previously reported and merits further investigation.

## Figures and Tables

**Table 1:** Characteristics of the study population.

	No.	Percent
Sex		
Male	134	38.1
Female	218	61.9
Age group (years)		
< 45	168	47.7
≥ 45	184	52.3
BMI category (kg/m^2^)		
< 25	57	16.2
25–29.9	126	35.8
≥ 30	169	48.0
Smoking status^a^		
Nonsmoker	90	25.6
Smoker	262	74.4
Glucose level (mg/dL)		
≤ 125	292	83.0
> 125	60	17.0
Self-reported diabetes		
Yes	65	18.5
No	287	81.5
Medication status		
On medication	303	86.1
Not on medication	49	13.9
Diabetes^b^		
Nondiabetic	281	79.8
Diabetic	71	20.2

a Having smoked > 100 cigarettes over lifetime.

b Based on either taking antidiabetic drugs or having a serum fasting glucose of ≥ 125 mg/dL.

**Table 2:** Distribution of serum glucose, age, BMI, and lipid measurements among study participants.

	Median	Mean ± SD	Range
Glucose (mg/dL)	94	110.3 ± 48.0	69–480
Age (years)	45.6	48.8 ± 13.2	30.1–84.8
BMI (kg/m^2^)	29.7	30.5 ± 6.4	15.7–59.8
Cholesterol (mg/L)	196	198.9 ± 38.0	101–306
Triglycerides (mg/L)	137	158.5 ± 95.2	41–746
Lipid (mg/L)^a^	659	672.4 ± 151.2	372.1–1416.7

a Estimated total lipid based on direct measurement of serum total cholesterol and triglycerides.

**Table 3:** Method detection limits (MDL) and the distribution of certain serum analyte concentrations before and after lipid standardization.

					Percentile			
Analyte	MDL	% > MDL	Mean ± SD	Min	33rd	50th	67th	Max
Wet-weight value (ppb)								
Total PCBs	NA	NA	5.03 ± 4.29	0.51	2.80	3.87	5.28	48.32
PCB-153	0.02	99.7	0.70 ± 0.61	0.00	0.39	0.52	0.74	6.68
PCB-74	0.02	98.9	0.33 ± 0.43	0.00	0.12	0.19	0.28	4.79
Mirex	0.02	86.4	0.13 ± 0.16	0.00	0.05	0.08	0.13	1.67
DDE	0.02	100.0	3.64 ± 3.66	0.14	1.60	2.42	3.50	22.15
HCB	0.02	97.7	0.08 ± 0.04	0.00	0.06^a^	0.07	0.09	0.33
Lipid-standardized value (ng/g lipid)								
Total PCBs	NA	NA	748.8 ± 635.6	84.8	448.6	579.8	756.2	7110.1
PCB-153	NA	NA	104.5 ± 91.2	0.0	59.8	78.3	104.4	982.9
PCB-74	NA	NA	49.0 ± 68.4	0.0	19.2	28.0	38.7	704.8
Mirex	NA	NA	19.2 ± 23.0	0.0	8.3	12.4	19.6	245.7
DDE	NA	NA	537.0 ± 512.6	20.3	246.1	349.5	544.6	3005.2
HCB	NA	NA	12.1 ± 6.5	0.0	9.1	11.1	13.0	61.9

Abbreviations: Max, maximum; Min, minimum; NA, not applicable.

a HCB had a very narrow distribution among participants in this study; 30%, 41%, 55%, 64% and 72% had a concentration ≤ 0.05 ppb, 0.06 ppb, 0.07 ppb, 0.08 ppb, and 0.09 ppb, respectively.

**Table 4:** Association between diabetes and serum concentrations of total PCBs, PCB-153, PCB-74, mirex, HCB, and DDE, adjusted for certain diabetes risk factors.^a^

	Wet-weight measurement		Lipid-standardized measurement	
Analyte	Unadjusted for the other analytes^b^ OR (95%CI)	Concurrent adjustment for the other analytes^b^ OR (95%CI)	Unadjusted for the other analytes^b^ OR (95%CI)	Concurrent adjustment for the other analytes^b^ OR (95%CI)
Total PCBs (ppb)				
Medium tertile	2.2 (0.8–5.9)	1.8 (0.6–5.5)	1.8 (0.8–4.3)	1.5 (0.6–4.0)
Highest tertile	3.9 (1.5–10.6)	2.8 (0.7–10.8)	3.2 (1.4–7.5)	2.6 (0.8–8.1)
Mirex (ppb)				
Medium tertile	1.2 (0.5–2.7)	0.7 (0.3–1.7)	0.8 (0.3–2.0)	0.6 (0.3–1.4)
Highest tertile	1.0 (0.4–2.2)	0.3 (0.1–0.8)	0.9 (0.4–2.2)	0.3 (0.1–0.9)
DDE (ppb)				
Medium tertile	1.8 (0.6–5.2)	1.4 (0.4–4.3)	2.4 (0.7–8.3)	1.6 (0.5–4.8)
Highest tertile	6.4 (2.2–18.4)	2.6 (0.8–8.8)	6.2 (1.8–21.9)	2.4 (0.7–8.3)
HCB (ppb)				
Medium tertile	0.9 (0.3–2.7)	0.9 (0.3–2.6)	2.7 (0.9–8.0)	2.5 (0.9–6.8)
Highest tertile	6.2 (2.3–16.9)	4.5 (1.4–14.3)	6.8 (2.3–20.3)	4.8 (1.7–13.9)
PCB-153 (ppb)				
Medium tertile	1.0 (0.4–2.5)	0.8 (0.2–2.4)	1.0 (0.4–2.3)	0.6 (0.2–1.6)
Highest tertile	3.2 (1.3–8.2)	3.0 (0.7–12.8)	2.4 (1.0–5.6)	1.4 (0.4–4.8)
PCB-74 (ppb)				
Medium tertile	1.3 (0.4–3.7)	1.3 (0.4–4.4)	1.3 (0.3–4.7)	0.9 (0.3–3.0)
Highest tertile	4.9 (1.7–13.7)	3.6 (1.0–13.4)	4.5 (1.3–15.6)	2.9 (0.8–10.5)

CI, confidence interval.

a All ORs were adjusted for sex, age category, BMI category, and lifetime smoking status; in addition, wet-weight values were adjusted for estimated total lipid concentration.

b Other analytes included serum concentrations of DDE, HCB, and mirex for total PCBs, PCB-153, and PCB-74; total PCBs, DDE, and HCB for mirex; total PCBs, mirex, and HCB for DDE; and total PCBs, mirex, and DDE for HCB.
